# Photosensitive Hydrogel-Based Embolic Agent Treatment of Wide-Necked Aneurysms: Preliminary Animal Results

**DOI:** 10.3390/gels8120788

**Published:** 2022-12-01

**Authors:** Jerry C. Ku, Yuta Dobashi, Christopher R. Pasarikovski, Joel Ramjist, Clement Hamani, Chinthaka Heyn, Konrad Walus, Victor X. D. Yang

**Affiliations:** 1 Division of Neurosurgery, Department of Surgery, University of Toronto, Toronto, ON M5T 2S8, Canada; 2 Institute of Medical Sciences, University of Toronto, Toronto, ON M5S 1A8, Canada; 3 Department of Electrical, Computer and Biomedical Engineering, Toronto Metropolitan University, Toronto, ON M5B 2K3, Canada; 4 Division of Neuroradiology, Department of Radiology, University of Toronto, Toronto, ON M5T 1W7, Canada; 5 Department of Electrical and Computer Engineering, University of British Columbia, Vancouver, BC V6T 1Z4, Canada; 6 Division of Neurosurgery, Department of Clinical Neurological Sciences, Schulich School of Medicine, Western University, London, ON N6A 3K7, Canada

**Keywords:** hydrogel, cerebral aneurysm, photomodulation, subarachnoid hemorrhage, animal model

## Abstract

**Background:** The endovascular treatment of cerebral aneurysms has become widespread but may still be limited by recurrence rates or complications. The discovery of novel embolic strategies may help mitigate these concerns. **Methods:** We formulated a Photosensitive Hydrogel Polymer (PHP) embolic agent which is low-viscosity, shear-thinning, and radio-opaque. After the filling of an aneurysm with PHP with balloon assistance, we utilized photopolymerization to induce solidification. Different methods of light delivery for photopolymerization were assessed in silicone models of aneurysms and in four acute animal trials with venous anastomosis aneurysms in pigs. Then, balloon-assisted embolization with PHP and photopolymerization was performed in three aneurysms in pigs with a one-month follow-up. Filling volume, recurrence rates, and complications were recorded. **Results:** The PHP was found to be suitable for the intravascular delivery and treatment of cerebral aneurysms. It was found that light delivery through the balloon catheter, as opposed to light delivery through the injection microcatheter, led to higher rates of filling in the 3D model and acute animal model for cerebral aneurysms. Using the balloon-assisted embolization and light delivery strategy, three wide-necked aneurysms were treated without complication. One-month follow-up showed no recurrence or neck remnants. **Conclusions:** We demonstrated a novel method of balloon-assisted photosensitive hydrogel polymer embolization and photopolymerization, leading to complete aneurysm filling with no recurrence at 1 month in three wide-necked aneurysms in pigs. This promising methodology will be investigated further with longer-term comparative animal trials.

## 1. Introduction

Intracerebral aneurysms are increasingly being treated endovascularly, due to the minimally invasive nature of this treatment compared to open surgery [1]. The most common endovascular treatment is the placement of metallic detachable coils into the aneurysm to promote thrombosis, which has been found to have lower rates of morbidity and mortality compared to surgical clipping [1,2,3]. Complete thrombosis within the aneurysm leads to a cure, but incomplete thrombosis can lead to aneurysmal recurrence. Recurrence after coiling is still observed in up to >30% of cases, with up to 20% requiring recoiling, leading to increased rates of retreatment or rebleeding compared to clipping [3,4]. Occlusion rates are related to aneurysm packing volume by the coils, where filling less than 20% often results in recurrence, but this must be balanced with excessive filling with coils which may lead to intra-procedural rupture [5,6,7,8]. Balloon or stent assistance may be required for wide-necked aneurysms [3]. Though coils were initially manufactured as bare platinum coils, there have been advancements in the use of additives or coatings to induce thrombosis or increase packing volume [9,10,11]. For example, Hydrocoils^®^ are platinum coils coated with hydrogel matrices which absorb water and swell in vivo, increasing overall packing volume [9]. Although rates of recurrence were decreased, the possibility of thromboembolism or delayed hydrocephalus remain a concern [12,13,14].

Other intrasaccular treatments for aneurysms include the Woven EndoBridge (WEB) device, or filling with liquid embolic agents. The WEB device, like with coils, provides a scaffolding for thrombosis to occur [15,16,17]. On the other hand, liquid agents may be utilized to ‘fill’ the aneurysm completely, without reliance on clotting. This has been argued to be able to lead to a more durable occlusion with decreased rates of recurrence [11,18,19,20]. For example, Onyx 500 can be used to treat aneurysms that are otherwise difficult to coil, namely, very large or very small aneurysms, irregularly shaped aneurysms, or recurrent previously coiled aneurysms [18]. However, even with balloon assistance, there are still some concerns including the risk of parent artery stenosis or occlusion due to encroachment of the Onyx into the artery, or of non-target embolization with improper balloon placement and/or insufficient time of inflation for Onyx solidification [18].

The discovery of novel embolic agents or materials may lead to further advancements in the care of cerebral aneurysms. A liquid agent with improved operator control may have the potential for filling aneurysms completely before solidifying, leading to decreased rates of recurrence. We have previously formulated a Photosensitive Hydrogel Polymer (PHP) for use in embolization procedures [21,22], which may also have potential in the treatment of cerebral aneurysms. The goal of this study was to test the photo-rheology of our novel liquid embolic agent via qualitative assessment in a 3D model of cerebral aneurysm, and then assess the preliminary safety and efficacy in its use in 3D phantom and animal models of wide-necked aneurysms, via evaluation of filling volumes, optical coherence tomography, and complication rates.

## 2. Results

### 2.1. 3D Model

**Figure 1:**
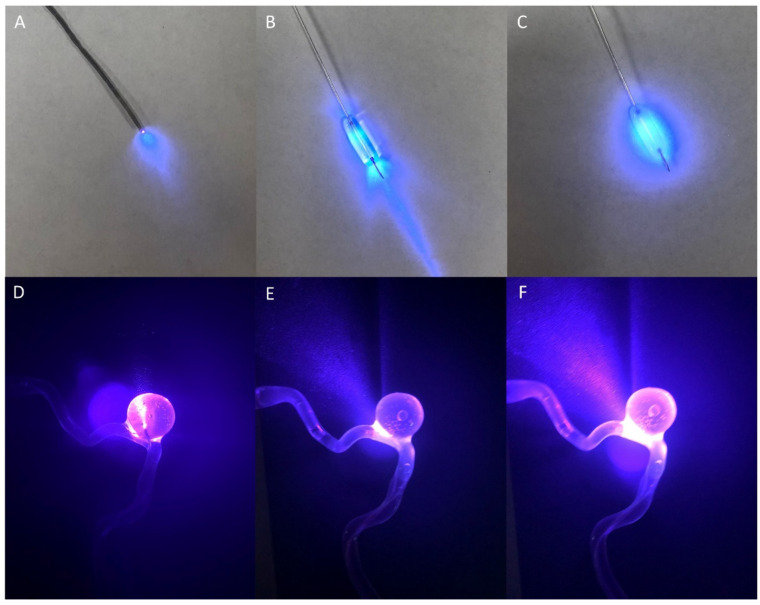
Light geometry associated with the different modes of light delivery, with the optical fiber threaded into the injection catheter (**A**), or into the balloon catheter inflated with contrast–saline (**B**) or contrast–saline–intralipid (**C**). Dark room view of the light irradiation within the model aneurysm presented with the injection catheter lighting (**D**) versus balloon catheter lighting without (**E**) or with (**F**) intralipid.

**Figure 2:**
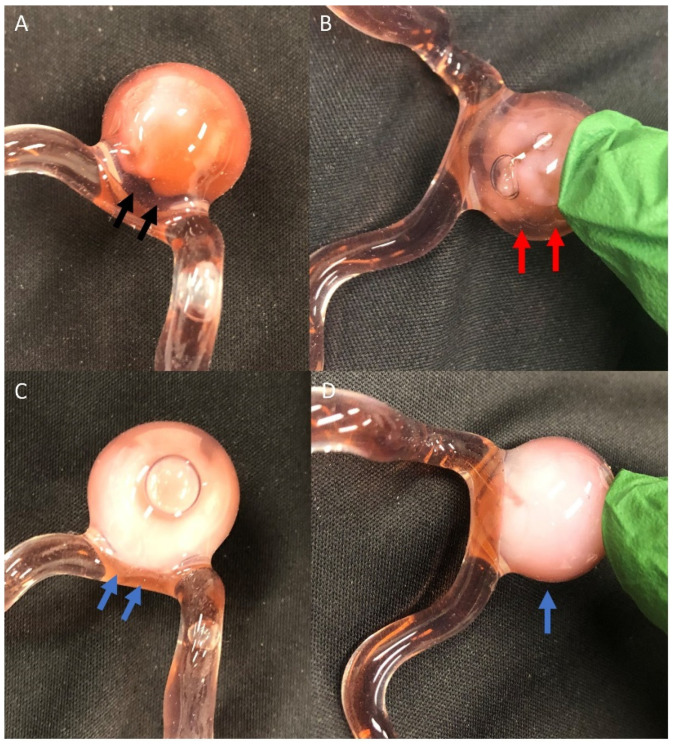
Photosensitive Hydrogel Polymer embolization of the model aneurysm using the injection catheter lighting (**A**,**B**) versus balloon lighting (**C**,**D**), with the black arrows demonstrating residual neck remnant of the aneurysm, the red arrows showing unfilled aneurysm at the edges after the injection catheter lighting, and the blue arrows showing complete filling of aneurysm, including the neck, using the balloon lighting.

In the silicone aneurysm model, we demonstrate the light geometries associated with the different irradiation strategies. As seen in Figure 1A and Figure 2C, placing the light source within the catheter (Injection Catheter Light; ICL) leads to strong light irradiation at the tip of the catheter and a cone of light emanating forward. On the other hand, placing the light source within the balloon (Balloon Lighting with Normal Saline; BLNS_ (Figure 1B,E) and Balloon Lighting with Intralipid (BLI) (Figure 1C,F) led to light irradiation being concentrated at the parent vessel over the neck of the aneurysm, with the addition of intralipid allowing for a more even light distribution and increased light irradiation into the aneurysm itself, reaching the dome.

In the pulsatile 3D aneurysm model, PHP injection is performed to fill the aneurysm with balloon assistance. The light source is then turned on to 20 mW to allow for photopolymerization for 3 min. The light is then turned off, the balloon deflated, and the injection and balloon catheters are removed. With the ICL method, areas of the PHP within the aneurysm are not fully polymerized, and thus some remain liquid and escape into the parent vessel. As seen in Figure 2A,B, this leads to an aneurysmal neck and areas of aneurysm remnant along the side wall of the aneurysm. On the other hand, BLNS or BLI lead to a full solidification of the PHP within the aneurysm, without any neck remnant (Figure 2C,D). 

**Figure 3:**
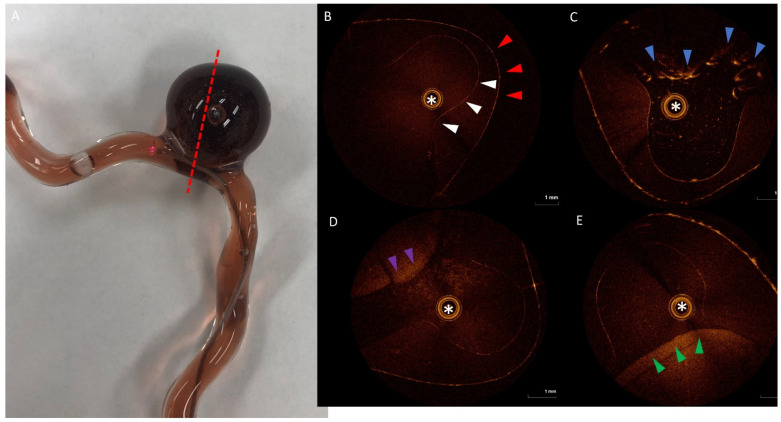
Cross-sectional OCT images of the model aneurysm (**A**) at the level of the dotted line. Pre-treatment OCT images demonstrate the parent vessel and the aneurysmal neck (**B**), with the white asterisk showing the imaging catheter, the white arrowheads showing the internal vessel wall of the model, and red arrowheads showing the external wall. The dome of the aneurysm is not visualized. After coiling (**C**), OCT shows the coil loops within the aneurysm (blue arrowheads) with a small gap in the neck area coverage. After PHP embolization using injection catheter lighting (**D**), there is a PHP mass (purple arrowheads) within the aneurysm but a gap between that mass and the aneurysm wall. After PHP injection using balloon lighting with intralipid (**E**), there is a PHP mass (green arrowheads) that fills the aneurysm and fully covers the aneurysm neck.

Using OCT, pre-embolization images of the silicone model were obtained for the parent vessel and for the aneurysm (see Figure 3). Given its large size, the aneurysm dome could not be visualized as the maximal imaging width of the OCT catheter is 10 mm; however, the aneurysmal neck could be imaged to a 10-micron resolution. After PHP treatment with the ICL method, OCT demonstrates the aneurysmal neck remnant that was suggested on macroscopic inspection of the model. On the other hand, after PHP treatment using the BLI method, a smooth and even layer of solidified PHP covers the aneurysm neck, without prolapse into the parent vessel. Comparatively, loops of coils at the neck of the aneurysm can also be visualized with OCT, with continued filling of the aneurysm between the coil interstices. 

### 2.2. Animal Model

**Figure 4:**
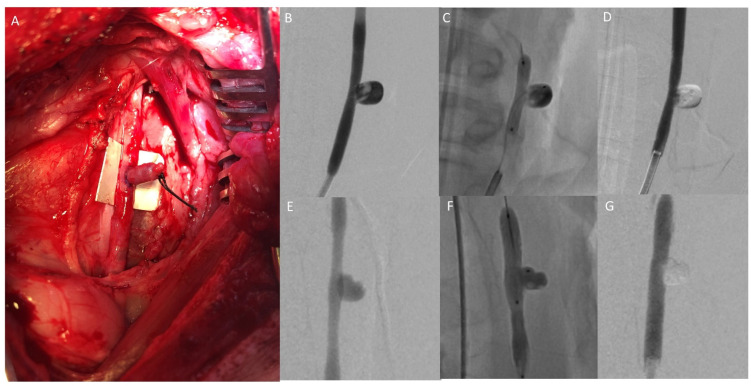
Venous pouch aneurysm in pig (**A**), with treatment using injection catheter lighting (**B**–**D**) or balloon lighting with intralipid (**E**–**G**). Pre-embolization angiography (**B**) showing the wide-necked aneurysm, fluoroscopy (**C**) showing the inflated balloon and PHP within the aneurysm, and post-embolization angiography (**D**) showing the PHP within the aneurysm and a small neck remnant. Pre-embolization angiography (**E**) showing the wide-necked aneurysm, fluoroscopy (**D**) showing the inflated balloon and PHP within the aneurysm, and post-embolization angiography (**F**) showing complete aneurysmal obliteration.

In the acute animal experiments (see Figure 4 and Table 1), injection catheter lighting led to <90% aneurysm filling with an obvious neck remnant in both cases, corresponding with an MRRC of II. In one case, there was also the off-target embolization of downstream external carotid artery branches. On the other hand, balloon lighting with intralipid led to >90% aneurysm filling with no neck remnant (MRRC I). 

**Figure 5:**
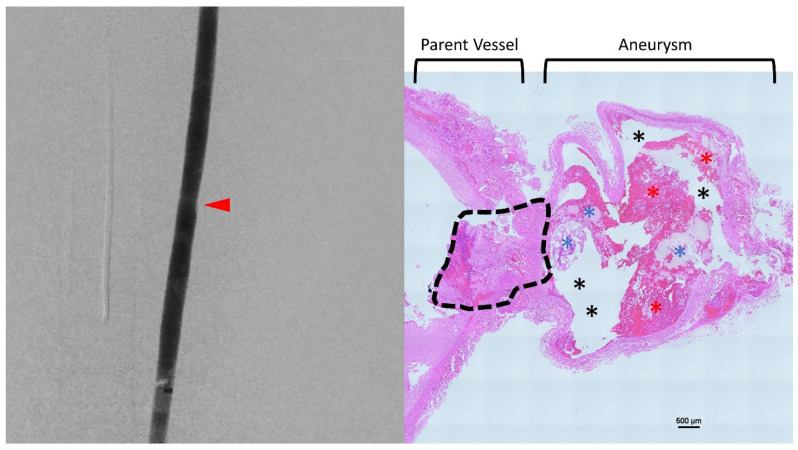
Follow-up angiography of the common carotid artery (**left**) showing complete obliteration of the aneurysm (red arrow). Histopathology (**right**) of the parent vessel and thrombosed aneurysm also shown, showing PHP (blue asterisks) and chronic thrombus (red asterisks) within the aneurysm, along with a preparation artefact (black asterisks) and robust endothelialization over the neck (within dotted line).

As such, in the subacute aneurysm experiments, BLI was utilized as the light delivery method. With balloon-assisted PHP embolization and light delivery, all three aneurysms were filled completely (MRRC I) with no residual/remnants and no complications (see Table 1). These findings were confirmed at four-week follow-up angiography (Figure 5-left). The specimens were harvested for histopathology (Figure 5-right), which revealed PHP and chronic thrombosis within the aneurysm and endothelialization over the neck of the aneurysm. It is noted that there was the partial washout and fragmentation of the PHP due to specimen processing, leading to an artefact within the aneurysm. There were no adverse findings in the parent vessel itself. 

## 3. Discussion

Herein, we demonstrate the utility of a novel liquid embolic agent using a Photosensitive Hydrogel Polymer (PHP) for the balloon-assisted treatment of wide-necked cerebral aneurysms. The base polymer is PEGDA, and nano-silicate platelets are added to impart shear-thinning behavior, allowing for the injection of higher viscosity material through neuro-interventional microcatheters [21,22]. The addition of Lithium phenyl-2,4,6-trimethylbenzoylphosphinate (LAP) allows for photo-activation with 405 nm light, leading to crosslinking between the PEGDA molecules, thus solidifying the hydrogel. Iodinated contrast is added to allow for visualization during the interventional radiology procedure. 

Liquid agents hold promise in increasing filling volume and thus decreasing recurrence or retreatment rates in cerebral aneurysms; however, they require balloon assistance to localize the embolic agent within the aneurysm while it solidifies [11,19]. While a currently available liquid embolic agent for this indication is Onyx 500, there remain clinical limitations with this agent. Some of the complications reported, such as parent artery encroachment, non-target embolization, or gluing or clogging of the catheter, are related to its self-solidification processes [11,18]. Other liquid embolic agents are commercially available, such as n-butyl cyanoacrylate (NBCA) glue, SQUID, and PHIL, but are not recommended for use in cerebral aneurysms [23,24,25]. 

There has been research interest in the development of other new liquid embolic agents that may address some of these concerns. Other authors have also sought to utilize hydrogels, a class of materials which are biologically inert, as investigational embolic agents [26,27,28], including for cerebral aneurysms [29,30,31]. The stimuli used to trigger solidification of a liquid hydrogel range from temperature [32,33], to pH [34], to chemical reactions, whether from a co-injected agent or due to a time-dependent reaction [27,29,35]. However, these processes may be difficult to control intra-procedurally and are relatively operator independent. On the other hand, light irradiation represents a method of polymerization that may allow for better spatiotemporal control, with the operator being able to ‘turn on the light switch’ [36,37]. This makes photopolymerization a promising embolization strategy for aneurysms. Recently, Lim et al. utilized photo-crosslinking at the tip of the catheter to spin hydrogel micro-fibers which were then ‘layered’ into a simple 3D model of cerebral aneurysms [38]. However, one specific advantage of a liquid embolization agent is the ability to completely ‘fill’ an aneurysm before solidification, and this may be lost with the aforementioned technique. Similarly, Poupart et al. injected hydrogel under balloon assistance with photopolymerization in in vitro silicone aneurysm models [30,39]. Both groups utilized an optical fiber placed within the wall of the injection microcatheter to transmit light from the laser source to the tip of the catheter [30,38]. 

On the other hand, one disadvantage of photopolymerization is the need to irradiate all of the injected polymer. Inactivated polymers remain in liquid form and may not stay localized within the vascular target once blood flow resumes. Even though small amounts may be biologically inert [30], this should still be avoided to prevent any possible unforeseen micro- or macro-embolic complications, or incomplete filling of the aneurysm. Herein, we demonstrate some of these concerns and how to best address them. 

With the optical fiber at the tip of the injection catheter, the light irradiation is forward facing in a conical shape. Any precursor that is at the level of the tip, the catheter, or ‘below’ the tip in the aneurysm may not be sufficiently irradiated and thus may not solidify and thus escape once the balloon is deflated, as seen in our silicone aneurysm model and acute animal model experiments. On the other hand, placing the optical fiber within the balloon allows for light irradiation over the neck of the aneurysm, while still being able to irradiate into the aneurysm, allowing for solidification over the important neck area to create a ‘seal’. This smooth surface of solidified PHP at the neck, as seen in OCT, may provide an attractive surface for endothelial cell growth, and thus aneurysmal healing. 

The addition of intralipid into the balloon catheter led to improved light scattering and a more even distribution of light irradiation. This is not surprising and is a well-known phenomenon that has been leveraged in other instances of light delivery in the medical field, such as in photodynamic therapy for tumors using balloon catheters [40,41,42]. An alternative to the above-described methods could be the addition of intralipid into the liquid polymer agent itself, which has been explored by Poupart et al. who found a small decrease in photopolymerization time and improved scattering, but this did not reach statistical significance [30]. We did not utilize this method due to concern that the addition of intralipid may change the embolic agent’s physical/rheological properties or biocompatibility profile. On the other hand, a concern with the balloon methodology of photo-crosslinking may be the irradiation of the parent vessel. However, intravascular light irradiation has already been previously utilized for the treatment of atherosclerosis or vasoconstriction without causing damage to endothelial cells or circulating cells within the bloodstream [43,44,45]. Moreover, histopathological analysis did not reveal damage to the endothelial cells following 5 min of 405 nm light irradiation at this intensity (20 mW). However, further analysis would still be required, including longer-term histopathological results. Additionally, as with other liquid embolic treatments, the use of a balloon catheter is required to localize the embolic agent in all aneurysm types, compared to coiling which does not require balloon assistance in simple saccular aneurysms. 

## 4. Conclusions

Our study has several limitations. The thin-walled vein of micro-anastomosed side-wall aneurysms does not fully approximate the histology of human cerebral aneurysms, compared to the elastase aneurysm model. Additionally, neither animal model can fully mimic human cerebral aneurysms, as they are often treated after creation and are not prone to rupturing [46]. However, this remains a well-established method for the creation of wide-necked aneurysms for preclinical testing [46,47,48], where novel treatments may first be tested for initial safety and feasibility [35]. There were only a small number of aneurysms treated, with only one-month follow-up. There was also no control group. Longer follow-ups and comparison with current gold-standard embolization treatment in animal models, such as coiling, will be required. 

In conclusion, we demonstrate a novel method of balloon-assisted photosensitive hydrogel polymer embolization and photopolymerization, leading to complete aneurysm filling in silicone aneurysm models and animal aneurysm models, with no recurrence at 1 month in three wide-necked aneurysms. This methodology will be investigated further with longer-term comparative animal trials. 

## 5. Materials and Methods

### 5.1. Materials

We have previously described the formulation and rheology of the PHP embolic agent, which is based on PEGDA (Sigma-Aldrich, St. Louis, MO, USA), nano-silicate platelets (BYK, Wesel, Germany), and lithium phenyl-2,4,6-trimethylbenzoylphosphinate (LAP; Sigma-Aldrich, St. Louis, MO, USA) as a photoinitator [21,22]. These components have been previously found to be biocompatible and non-cytotoxic in previous studies [26,29,49]. The components were sterile-filtered, and this methodology was chosen to prevent differences in rheological and physicochemical properties before and after sterilization. This was mixed with a radio-opaque contrast medium (Omnipaque 300, GE Healthcare, Chicago, IL, USA). Unlike other liquid embolic agents, the PHP requires light stimulation to polymerize and solidify. The light intensity can be modulated to fine-tune the photo-crosslinking and thus the extruded product [21,22]. 

### 5.2. Novel Embolic Agent and Delivery Strategy

**Figure 6:**
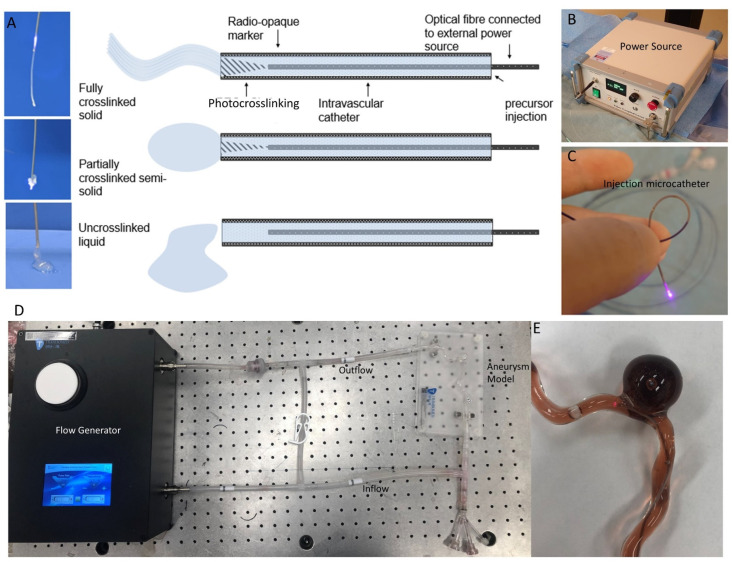
Pictorial representation of the different deposited forms of PHP based on variable photopolymerization (**A**) and the power source (**B**) with the injection microcatheter (**C**). The 3D-printed silicone with the pulsatile flow generator is shown (**D**) with a close-up view of the aneurysm model filled with circulating fluid (**E**).

Light is delivered from a 100 mW 405 nm laser source (CNI laser, Changchun, China) through a 100 μm optical fiber (AFM100H, Thorlabs, Newton, NJ, USA) to the site of the desired photopolymerization, as seen in Figure 6. For cerebral aneurysms, the PHP is deposited into the aneurysm through an injection catheter, while a second catheter with a balloon is inflated over the aneurysmal neck to keep the PHP localized within the aneurysm. After photo-solidification, the balloon can be deflated and both catheters removed. 

The two methods of light delivery to the PHP deposited within the aneurysm are (1) Injection-Catheter-Light (ICL) where the optical fiber is placed co-axially within a commercially available 1.7F neuro-interventional microcatheter (Excelsior^®^ SL10, Stryker, Kalamazoo, MI, USA) to deliver light at the tip of the microcatheter, which is also used for embolic agent injection, and (2) Balloon-Light (BL) where the optical fiber is placed co-axially within a 2.8F single-lumen balloon microcatheter (Hyperform^®^ 4 × 20 mm, Medtronic, Minneapolis, MIN) with a sealed distal tip to deliver light into the inflated balloon. The balloon may be inflated with (2a) Balloon-Light-Normal-Saline (BLNS): 50:50 iohexol and normal saline or (2b) Balloon-Light-Intralipid (BLI): 50:49 iohexol and normal saline with the addition of 1% intralipid for light scattering.

### 5.3. 3D Model Testing with OCT

The three methods of light delivery were visualized in a 3D-printed silicone model of a wide-necked cerebral aneurysm (see Figure 6) with a pulsatile flow generator (Trandomed, Ningbo, China), using circulating water dyed with red food coloring and India ink to mimic the light absorption of blood. The circulating fluid was warmed to 37 degrees Celsius to mimic real-life conditions. The model aneurysm is 15 mm × 15 mm with a 5 mm neck. The three methods of light delivery (ICL, BLNS, BLI) were tested, and aneurysm filling was estimated. For comparison, coiling of the model aneurysm was also performed. Optical Coherence Tomography (OCT) was deployed as a method of intravascular high-resolution imaging [50] to assess the surface of the PHP and any microscopic prolapses or aneurysmal neck remnants. OCT images were captured before and after aneurysm treatment, using a Dragonfly catheter (St. Jude Medical, Minneapolis, MN, USA) with the ILUMIEN OPTIS system (St. Jude Medical, Minneapolis, MN, USA).

### 5.4. Animal Model Testing

All experiments were conducted according to the policies and standards established by the authors’ institutional animal research ethics board (Sunnybrook Health Sciences Center Research Ethics Board). Yorkshire swine weighing 40–45 kg were utilized. All procedures were performed with the animal under a general anesthetic with continuous hemodynamic monitoring. A dedicated animal hybrid operating theatre equipped with a single-plane C-arm (Philips, Andover, MA, USA) was used for all procedures.

Prior to aneurysm creation, an ultrasound-guided 8F femoral puncture was performed, and an 8F guide catheter (Neuron Max 088, Penumbra, Alameda, CA, USA) was navigated to the common carotid artery. A wide-necked aneurysm was then created on the left common carotid artery using the venous pouch anastomosis method. In brief, a 1 cm portion of the left external jugular vein was harvested. The left common carotid artery was isolated at the mid-cervical region, temporary clips were applied, and a 5 mm arteriotomy was performed on the anterolateral wall. The venous pouch was then connected end-to-side to the arteriotomy with running 8-0 Prolene sutures (Ethicon, Cincinnati, OH, USA). The temporary clips were then released, and blood flow into both the aneurysm and parent artery were confirmed via catheter angiography. The size of the aneurysm was recorded. The incision was then closed in multi-layer fashion. 

The injection microcatheter was then navigated into the aneurysm, and the balloon catheter was navigated into the carotid artery covering the neck of the aneurysm and inflated. The injection of PHP was performed to fill the aneurysm, then the light was turned on to allow for photopolymerization for 3 min. The balloon catheter was then deflated (total balloon time 5 min). The injection and balloon catheters were then removed, and post-embolization angiography was performed.

In two acute aneurysm experiments, the optical fiber was threaded into the injection microcatheter until it was tip-to-tip with the microcatheter (ICL method). For two other animals, the optical fiber was threaded into the balloon catheter and the balloon catheter inflated with contrast and normal saline with 1% intralipid (BLI method). The degree of aneurysm occlusion was estimated based on fluoroscopy and post-embolization angiography using a reworked Modified Raymond–Roy Classification [51], where Class I = complete obliteration, Class II = residual neck, Class IIIa = contrast between the PHP layers of the aneurysm, and Class IIIb = contrast along the aneurysm wall. Total treatment time, amount of embolic material used, any instances of system failure, and other complications or aneurysm neck remnants or recurrence were recorded. 

In the three subacute aneurysm experiments, the optimal method of light delivery, as seen with the modeling and the acute testing, was utilized. Following treatment, the catheters were removed and an 8F Angio-Seal was used to close the femoral artery access. The animal was monitored for clinical or neurologic deficits. At four weeks, repeat angiograph was performed to assess for aneurysmal remnants, recurrence, or any other complications. After animal sacrifice, the segment of the carotid artery and aneurysm was harvested for histopathological examination. 

## Figures and Tables

**Table 1:** Acute and subacute animal model aneurysm results with PHP embolization.

Animal	Size of Aneurysm (Height × Width), Neck Width (mm)	Light Irradiation Method	MRRC	Complications
Acute experiments:				
1	Aneurysm size 9 × 7 mm, neck width 6 mm	ICL	II	None
2	8 × 6, 5	ICL	II	Non-target embolization
3	6 × 7, 7	BLI	I	None
4	8 × 7, 5	BLI	I	None
Subacute experiments:				
5	10 × 6, 5	BLI	I	None
6	8 × 5, 5	BLI	I	None
7	11 × 7, 6	BLI	I	None
